# Self-(in)compatibility genotypes of Moroccan apricots indicate differences and similarities in the crop history of European and North African apricot germplasm

**DOI:** 10.1186/1471-2229-13-196

**Published:** 2013-12-01

**Authors:** Ossama Kodad, Attila Hegedűs, Rafel Socias i Company, Júlia Halász

**Affiliations:** 1 Département d’Arboriculture, École Nationale d’Agriculture de Meknès, BP S/40, Meknès, Morocco; 2 Department of Genetics and Plant Breeding, Corvinus University of Budapest, P.O. Box 53, Budapest, H-1518, Hungary; 3 Unidad de Fruticultura, Centro de Investigación y Tecnología Agroalimentaria de Aragón (CITA), Av. Montañana 930, Zaragoza 50059, Spain

**Keywords:** Apricot, Crop evolution, Genetic variability, *S*-alleles, Self-incompatibility

## Abstract

**Background:**

Allelic diversity of the *S*-locus is attributed to the genetic relationships among genotypes and sexual reproduction strategy. In otherwise self-incompatible *Prunus* species, the emergence of loss-of-function in *S*-haplotypes has resulted in self-compatibility. This information may allow following major stages of crop history. The genetic diversity in the *S*-locus of local apricots (*Prunus armeniaca* L.) from different oasis ecosystems in Morocco and the comparison of the occurrence and frequency of *S*-alleles with other regions may allow testing the validity of previous theories on the origin and dissemination of North African apricots.

**Results:**

The *S*-genotypes of 55 Moroccan apricot accessions were determined, resulting in 37 self-compatible genotypes, from which 33 were homozygotes for self-compatibility. *S*_C_ was the most frequent *S*-allele in this germplasm, followed by *S*_13_, *S*_7_, *S*_11_, *S*_2_, *S*_20_, *S*_8_, and *S*_6_. New approaches (CAPS or allele-specific PCR) were designed for a reliable verification of the rare or unexpected alleles. The frequency and distribution of the *S*-alleles differed among the oases. Some of these alleles, *S*_8_, *S*_11_, *S*_13_ and *S*_20_, were formerly detected only in the Irano Caucasian germplasm and are not present in Europe.

**Conclusions:**

Our data supports the Irano-Caucasian origin of the Moroccan apricots and their original introduction by Phoenicians and Arabs through the North African shore. North Africa seems to have preserved much higher variability of apricot as compared with Europe. The loss of genetic diversity in apricot might be explained by the occurrence of self-compatibility and the length of time that apricot has spent with this breeding system in an environment without its wild relatives, such as the Moroccan oases or Central Europe.

## Background

Apricot (*Prunus armeniaca* L.) originated in Central Asia and was primarily domesticated in China, with a secondary centre of origin in the Middle East [1]. It is a traditional fruit crop in North Africa and its first introduction in the Maghreb is attributed to the Phoenicians more than 2500 years ago [2] due to the exchange of commercial products between the eastern and western parts of the Mediterranean basin. Evidence of its cultivation in the Maghreb can be traced back as far as to the 12^th^ century [3]. Apricot is supposed to have been again introduced in Morocco by the Arabs [4], belonging to a homogenous Maghreb gene pool, since Algerian, Moroccan and Tunisian apricot accessions have close genetic relationships [5]. Moroccan apricot material can be divided into two categories: local genotypes propagated by seed and recently introduced cultivars propagated by grafting, mainly ‘Canino’ and ‘Del Patriarca’. The introduced cultivars are grown according to a semi-intensive system, mainly in Central-South Morocco (Marrakech and its surrounding regions), whereas local genotypes are grown in oases and valleys in the South and Central-East Morocco according to the traditional system, without pruning, training and chemical treatments against pests and diseases [6].

Apricot belongs to the Rosaceae family and shows gametophytic self-incompatibility (GSI), a genetically controlled mechanism enabling styles to reject self-pollen [7]. Self-incompatibility is a mechanism of hermaphrodite plants to prevent self-fertilization and to promote out-breeding by inhibiting pollen tube growth within the third quarter of the style. Self-incompatibility in apricot is controlled by a single locus with multiple variants, called *S-*haplotypes [7]. The *S*-haplotype contains a gene encoding for a ribonuclease enzyme in the pistil, *S-*RNase [8], and an *S*-*haplotype-specific F-box* gene in the pollen [9,10]. The molecular basis of GSI is similar in the Solanaceae and Scrophulariaceae [11].

Much progress has been recently made in the identification of the *S*-genotypes in apricot. Seven *S*-alleles have been described in North American and Spanish apricot for self-incompatibility, labelled as *S*_1_-*S*_7_, and one for self-compatibility, identified as *S*_C_[12]. These alleles were later confirmed using a PCR approach [13]. Nine new alleles (*S*_8_-*S*_16_) were found among 27 apricot accessions from Hungary using non-equilibrium pH gradient electrofocusing (NEpHGE) and PCR [14]. Ten new putative alleles (*S*_11_-*S*_20_) were found among 16 native Chinese apricots with PCR and sequencing [15], although these alleles were not named following Halász et al. [14]. More recently, eight new *S*-alleles (*S*_23_-*S*_30_) have been identified and characterized in Chinese apricots using PCR and DNA sequencing [16]. The identification of *S*-alleles in different apricot cultivars is useful to establish cross-incompatibility groups to avoid pollination problems in the orchard and also to provide useful information for breeders to choose parental genotypes. From this information, 14 cross-incompatibility groups have been established for the North American, European and Turkish apricot cultivars [17-20]. Moreover, the determination of the *S*-alleles in different Turkish and Hungarian apricot accessions furnished molecular evidence supporting the historical connection between these two apricot germplasm [17]. Consequently, *S*-genotyping is also useful in the elucidation of crop evolution and propagation history of a given species.

Self-incompatibility is a common feature among apricot cultivars of the Central Asian and Irano-Caucasian eco-geographical groups, whereas the European group is traditionally considered to be self-compatible [21]. Central Asian and Chinese apricots have been shown to be self-incompatible using self-pollen tube growth observation under fluorescence microscopy, fruit set after self-pollination under field conditions, PCR and sequencing approaches [15,16,22]. However, the presence of self-compatibility has been confirmed for the apricot cultivars of the European and Irano-Caucasian groups [14,17,18]. Two mutations have been proposed to explain self-compatibility in these apricot groups. One has been the presence of the *S*_C_-allele [23,24]. The *S*_C_-haplotype has been confirmed to be a pollen-part mutant of the *S*_8_-haplotype [24], with a 358 bp insertion in the *SFB*_C_ gene [25]. This results in a truncated protein that lacks the hypervariable region that has a crucial role in the allele-specific recognition process [26]. The loss of the pollen *S* function was further supported by the identification of the original, non-mutated form of the apricot *SFB*_C_-allele in Hungarian and Turkish apricots [17,24]. Another mutation has been suggested outside the *S*-locus or by the presence of modifying factors. The PCR-analysis of progenies derived from ‘Canino’ (*S*_C_*S*_2_) showed that pollen grains carrying the *S*_2_-haplotype were also able to overcome the incompatibility barrier while coding sequence and expression pattern of the *SFB*_*2*_ and *S*_*2*_*-RNase* genes in ‘Canino’ were identical with those of the self-incompatible ‘Goldrich’ (*S*_1_*S*_2_) [25].

As far as we know there are no studies concerning the *S*-allele diversity in the southern Mediterranean apricot group, which is considered to belong to the Irano-Caucasian gene pool [4]. Thus, our main objective was to evaluate the genetic diversity in the *S*-locus of local apricots from different oasis ecosystems in Morocco. The occurrence and frequency of *S*-alleles in this region could be compared to those of other regions in order to support previous theories on the origin of North African apricots, as well as to examine the genetic consequences of the modification in the sexual reproduction strategy of fruit trees.

## Results and discussion

### Identification of S-alleles

**Figure 1:**
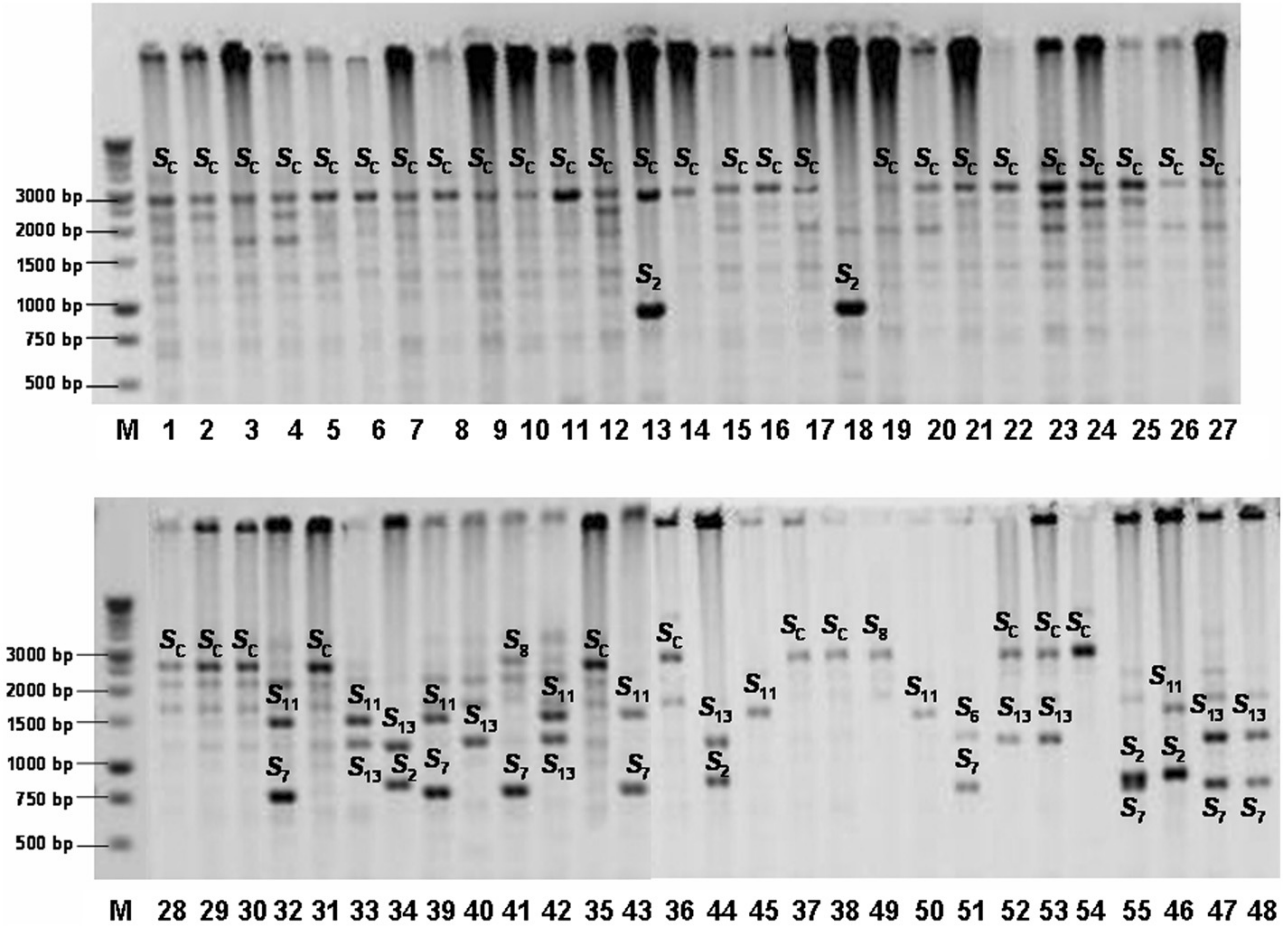
**PCR products (in negative) in 55 Moroccan apricot accessions using the second intron consensus primers of *Prunus S*-*RNase *gene.** M: 1 kb?+?DNA ladder; numbers refer to samples shown in Table 1.

Determination of the *S*-genotypes of 55 Moroccan apricot accessions was carried out using the consensus primers for the first and second introns of the *S*-*RNase* gene. The sizes of the PCR products obtained were compared with those previously published [13,27]. PCR amplification of genomic DNA with the consensus primers designed from conserved coding regions flanking the second intron of apricot *S*-*RNases*, yielded two fragments of various sizes in 12 genotypes, just one band in 40 genotypes, and no fragment in three genotypes (Figure 1; Table 1).

**Table 1:** **Label, site of origin, sizes of the 1**^**st **^**and 2**^**nd **^**intron regions of the *S-RNase *** **gene, specific PCR for the *S***_**C/8**_**-*RNase *** **and *SFB***_**C/8 **_**alleles and *S*-genotypes of the tested Moroccan apricots**

**Number**	**Sample**	**Site**	**1**^**st **^**intron (bp)**	**2**^**nd **^**intron (bp)**	***S***_**C**_**/*S***_**8**_**-*RNase***	**FB**_**C/8**_	***S*-genotype**
1	Armed-22	Agdz	355	2800	+	*S*_C_	*S*c*S*c
2	Armed-20	Agdz	355	2800	+	*S*_C_	*S*c*S*c
3	Armed-6	Agdz	355	2800	+	*S*_C_	*S*c*S*c
4	Armed-5	Agdz	355	2800	+	*S*_C_	*S*c*S*c
5	Armed-16	Agdz	355	2800	+	*S*_C_	*S*c*S*c
6	Armed-15	Agdz	355	2800	+	*S*_C_	*S*c*S*c
7	Armed-18	Agdz	355	2800	+	*S*_C_	*S*c*S*c
8	Tighnit-11	Agdz	355	2800	+	*S*_C_	*S*c*S*c
9	Tighnit-19	Agdz	355	2800	+	*S*_C_	*S*c*S*c
10	Tighnit-7	Agdz	355	2800	+	*S*_C_	*S*c*S*c
11	Tighnit-8	Agdz	355	2800	+	*S*_C_	*S*c*S*c
12	Tighnit-13	Agdz	355	2800	+	*S*_C_	*S*c*S*c
13	Tighnit-4	Agdz	355	950, –	+	*S*_C_	*S*c*S*_2_
14	Tighnit-14	Agdz	355	2800	+	*S*_C_	*S*c*S*c
15	Tighnit-1	Agdz	355	2800	+	*S*_C_	*S*c*S*c
16	Tighnit-20	Agdz	355	2800	+	*S*_C_	*S*c*S*c
17	Tighnit-16	Agdz	355	2800	+	*S*_C_	*S*c*S*c
18	Tighnit-5	Agdz	223, 332	950, –	–	–	*S*_2_*S*_20_
19	Armed-11	Agdz	355	2800	+	*S*_C_	*S*c*S*c
20	Tighnit-6	Agdz	355	2800	+	*S*_C_	*S*c*S*c
21	Tighnit-2	Agdz	355	2800	+	*S*_C_	*S*c*S*c
22	Tighnit-18	Agdz	355	2800	+	*S*_C_	*S*c*S*c
23	Tighnit-15	Agdz	355	2800	+	*S*_C_	*S*c*S*c
24	Tighnit-21	Agdz	355	2800	+	*S*_C_	*S*c*S*c
25	Tighnit-12	Agdz	355	2800	+	*S*_C_	*S*c*S*c
26	Tighnit-3	Agdz	355	2800	+	*S*_C_	*S*c*S*c
27	Tighnit-23	Agdz	355	2800	+	*S*_C_	*S*c*S*c
28	Tighnit-17	Agdz	355	2800	+	*S*_C_	*S*c*S*c
29	Tighnit-9	Agdz	355	2800	+	*S*_C_	*S*c*S*c
30	Tighnit-22	Agdz	355	2800	+	*S*_C_	*S*c*S*c
31	Tighnit	Agdz	355	2800	+	*S*_C_	*S*c*S*c
32	Rich-2	Atlas mount.	305, 401	750, 1500	–	–	*S*_7_*S*_11_
33	Rich-1	Atlas mount.	289, 305	1350, 1500	–	–	*S*_11_*S*_13_
34	Rich	Atlas mount.	332, 379	950, 1350	–	–	*S*_2_*S*_13_
35	Lahcoun-2	Skoura	355	2800	+	*S*_C_	*S*c*S*c
36	Lahcoun-1	Skoura	355	2800	+	*S*_C_	*S*c*S*c
37	Skoura-5	Skoura	355	–	+	*S*_C_	*S*c*S*c
38	Skoura-4	Skoura	355	–	+	*S*_C_	*S*c*S*c
39	Ait-Lhcen-7	Kelaat M’Gouna	305, 401	750, 1500	–	–	*S*_7_*S*_11_
40	Ait-Lhcen-6	Kelaat M’Gouna	223, –	–, 1350	–	–	*S*_13_*S*_20_
41	Ait-Lhcen-3	Kelaat M’Gouna	355, 401	2800, 750	+	*S*_8_	*S*_7_*S*_8_
42	Ait-Lhcen-4	Kelaat M’Gouna	289, 305	1350, 1500	–	–	*S*_11_*S*_13_
43	Ait-Lhcen-2	Kelaat M’Gouna	305, 401	750, 1500	–	–	*S*_7_*S*_11_
44	Ait-Lhcen-1	Kelaat M’Gouna	332, 379	950, 1350	–	–	*S*_2_*S*_13_
45	Ait-Lhcen-5	Kelaat M’Gouna	223, 305	–, 1500	–	–	*S*_11_*S*_20_
46	Ait-Talat-3	Kelaat M’Gouna	306, 332	950, 1500	–	–	*S*_2_*S*_11_
47	Ait-Talat-1	Kelaat M’Gouna	379, 401	1350, 750	–	–	*S*_7_*S*_13_
48	Ait-Talat-5	Kelaat M’Gouna	379, 401	1350, 750	–	–	*S*_7_*S*_13_
49	Gulmim-2	Gulmima	223, 355	–, 2800	+	*S*_8_	*S*_8_*S*_20_
50	Gulmim-5	Gulmima	223, 306	–, 1500	–	–	*S*_11_*S*_20_
51	Gulmim-8	Gulmima	424, 401	1300, 750	–	–	*S*_6_*S*_7_
52	Gulmim-3	Gulmima	355, 379	–, 1350	+	*S*_C_	*S*c*S*_13_
53	Gulmim-7	Gulmima	355, 379	–, 1350	+	*S*_C_	*S*c*S*_13_
54	Gulmim-1	Gulmima	355, 223	–	+	*S*_C_	*S*c*S*_20_
55	Gulmim-4	Gulmima	332, 401	950, 750	–	–	*S*_2_*S*_7_

**Figure 2:**
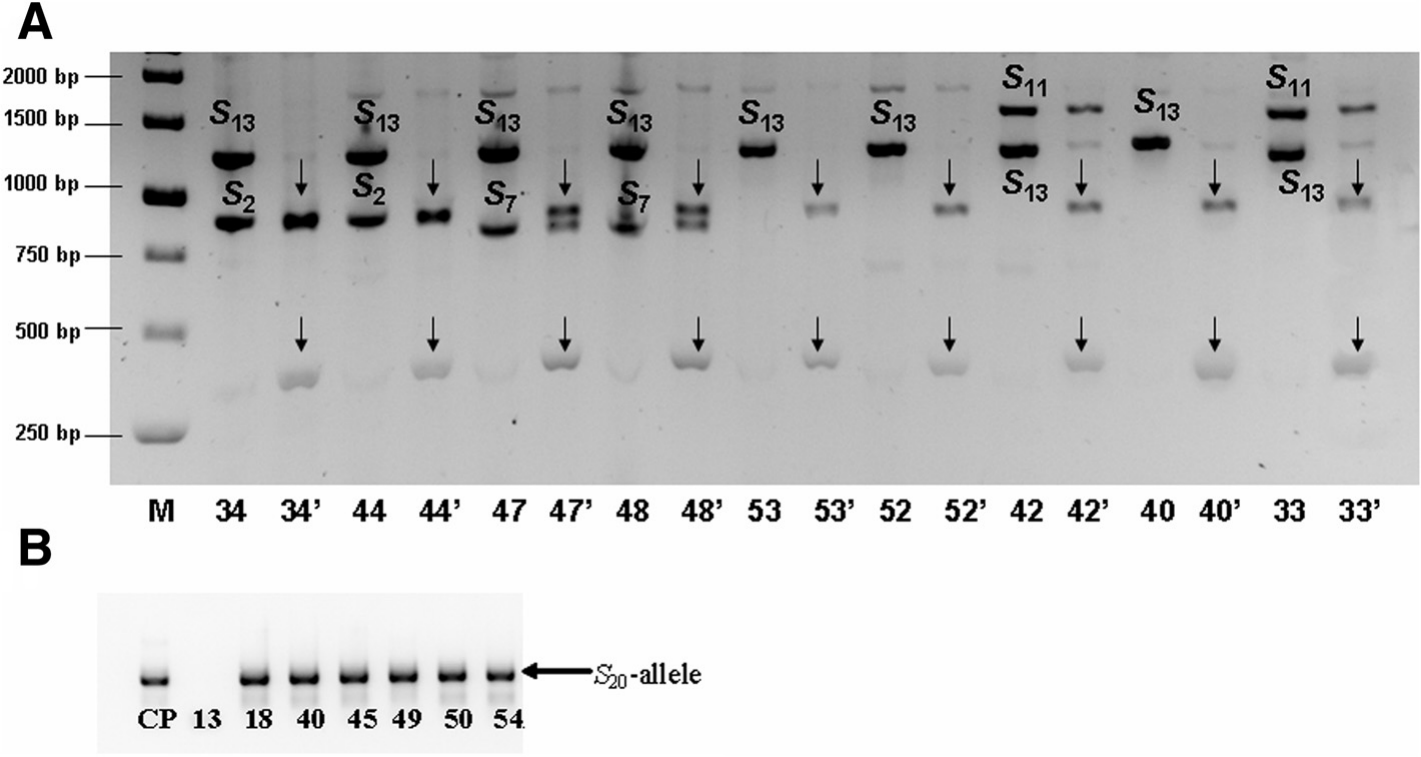
**Confirmation of the rare or unexpected alleles in Moroccan apricot accessions. (A)***S*_13_ allele-specific digestion with the enzyme *Pst*I and **(B)** the *S*_20_ allele-specific primer. CP: ‛Ceglédi Piroska’ (*S*_8_*S*_20_), numbers refer to samples shown in Table 1.

The fragment sizes of the second intron region ranged from 750 to 2800 bp (Table 1). In the genotypes Tighnit-4, Tighnit-5, Rich, Ait-lahcen-1, Ait-talat-3 and Gulmima-4, a fragment of approx. 950 bp was detected, which indicates the presence of the allele *S*_2_. In Rich-2, Ait-lahcen-2, Ait-lahcen-3, Ait-lahcen-7, Ait-talat-1, Ait-talat-5, Gulmima-4 and Gulmima-8 a fragment of approx. 750 bp was detected, which indicates that they share the *S*_7_-allele. The allele *S*_11_ was amplified in 8 genotypes (Rich-1, Rich-2, Ait-Lahcen-2, Ait-Lahcen-4, Ait-Lahcen-5, Ait-Lahcen-7, Ait-Talat-3 and Gulmima-5) as a fragment of approx. 1500 bp. The *S*_13_-allele was detected in 10 genotypes (Rich, Rich-1, Ait-Lahcen-1, Ait-Lahcen-4, Ait-Lahcen-6, Ait-Lahcen-7, Ait-Talat-1, Ait-Talat-5, Gulmima-3 and Gulmima-7) as a fragment of approx. 1350 bp. The presence of the *S*_13_-allele was confirmed by digestion of the second intron PCR fragments using *Pst*I enzyme (Figure 2A). The enzyme specifically cut the second intron PCR product into two fragments of 356 and 994 bp. A unique band size of 2800 bp was detected in 34 genotypes (Figure 1; Table 1), which indicates the possible presence of the *S*_8_- or *S*_C_-allele. The use of the degenerate primers EM-PC2consFD and EM-PC3consRD [28] for the amplification of the second intron region of the *S-RNase* gene did not allow the identification of one of the two *S*-alleles in several Moroccan genotypes. Similar results were reported by Halász et al. [17] when evaluating the *S*-alleles in Turkish genotypes. This situation may be due to homozygosity for the *S*_C_-allele or to preferential PCR amplification probably due to significant differences in the GC content of the two alleles, or in the DNA adjacent to these sequences [29].

The first intron lengths have been useful to confirm the identity of the *S*-alleles in apricot [17]. Hence, the first intron lengths were determined using fluorescently labelled primers and automated sizing. Sizes of the first intron regions ranged between 223 bp and 424 bp (Table 1). Two bands were amplified in 20 genotypes and only one fragment of 355 bp in 35 genotypes. Combining the band sizes of the first and second introns and comparing them with previous studies [13,17], the band size of 332 bp was attributed to *S*_2_; that of 401 bp to *S*_7_, that of 306 bp to *S*_11_, and that of 379 bp to *S*_13_ (Table 1). In the genotype Gulmim-8, a fragment of approx. 1400 bp was yielded for the second intron (Figure 1), which was only slightly different from the size of the *S*_13_-allele. However, a band size of 424 bp was detected for the first intron, which makes a real difference compared to the size of 379 bp of *S*_13_ and characteristic for the *S*_6_-allele. Thus, we have assigned the *S*_6_-allele to this genotype based on the combination of length sizes of the two introns. According to Halász et al. [17], the first intron size of the *S*_20_-allele is 222 bp, which has been detected in 6 genotypes (Gulmim-1, Gulmim-2, Gulmim-5; Ait lahcen-5; Ait lahcen-6; Tighnit-5). In these genotypes, no fragment was amplified using the degenerate primers EM-PC2consFD and EM-PC3consRD [28] for the amplification of the second intron region. To selectively detect the *S*_20_-allele, an allele-specific forward primer was designed and used in combination with the reverse EM-PC5consRD primer [28]. Genotypes sharing the *S*_20_-allele amplified a fragment of approx. 600 bp (Figure 2B), similarly to the Hungarian apricot ‘Ceglédi Piroska’ used as a control [27]. In other genotypes, there was no amplification.

**Figure 3:**
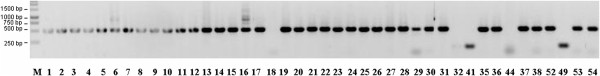
**PCR amplification of the *SFB *gene to differentiate between *SFB***_**C **_**and *SFB***_**8 **_**alleles in Moroccan apricots.** M: 1 kb?+?DNA ladder; numbers refer to samples shown in Table 1. Samples 18 (*S*_2_*S*_20_), 32 (*S*_7_*S*_11_) and 44 (*S*_2_*S*_13_) were used as negative controls to indicate the reliability of the *S*_C_/*S*_8_-haplotype-specific PCR.

A fragment of 355 bp was detected in 39 genotypes (Table 1). This fragment size was previously attributed to the *S*_C_- and *S*_8_-*RNase* alleles [24]. Combining these results with those obtained using the primers EM-PC2consFD and EM-PC3consRD [28] for the amplification of the second intron region; we determined either the *S*_C_- or *S*_8_-allele in 39 genotypes (Figure 1; Table 1). In 6 genotypes (Gulmima-1, Gulmima-3, Gulmima-7, Tighnit-4, Skoura-4 and Skoura-5), no amplification was yielded for the second intron to confirm the presence of these alleles. We used a specific primer (AprSC8) designed by Halász et al. [17] to anneal exclusively within the second intron region of the *S*_C_- and *S*_8_-*RNase* alleles. This primer pair amplified a fragment of 547 bp (data not shown) attributed to *S*_8_/*S*_C_-alleles by Halász et al. [17]. These results confirmed definitively the presence of the *S*_8_/*S*_C_-alleles in the tested 39 Moroccan genotypes. Since coding regions of the *S*_8_- and *S*_C_-*RNase* alleles are identical, discrimination of SI from SC cultivars could not be achieved in this analysis. The *S*_8_- and *S*_C_-haplotypes differ only in the *SFB* gene with an insertion of 358 bp found in the *SFB*_C_ and resulting in a truncated protein and the consequent breakdown of self-incompatibility [25]. Using a primer pair (AprFBC8) [17], we distinguished between the *S*_8_- and *S*_C_-haplotypes since accessions carrying the *SFB*_C_-allele showed an amplification product of approx. 500 bp size, while genotypes carrying the *SFB*_8_-allele showed a fragment of approx. 150 bp (Figure 3). Based on the results, 37 genotypes proved to be self-compatible carrying the *S*_C_-haplotype, from which 33 were homozygous and only four were heterozygous (Table 1). The *S*_8_-allele was identified in only two genotypes (Figure 3).

The self-(in)compatibility phenotype was tested in 19 genotypes by determining fruit set percentage and following pollen tube growth after self-pollination [30], showing that Rich-1, Rich-2, Gulmim-2, Gulmim-4 and Gulmim-5 are self-incompatible. The present results confirmed that these genotypes did not harbour the *S*_C_-allele (Table 1). However, the *S*_C_-allele was present in the other 14 genotypes (Table 1), confirming their self-compatible phenotype [30]. Thus, it appears that the expression of self-compatibility in the Moroccan apricot germplasm is also due to the presence of the *S*_C_-allele, as reported for other apricots from different countries including Hungary [14], Turkey [17] and Spain [13].

### S-allele frequency and distribution among the oasis agro-ecosystems

**Figure 4:**
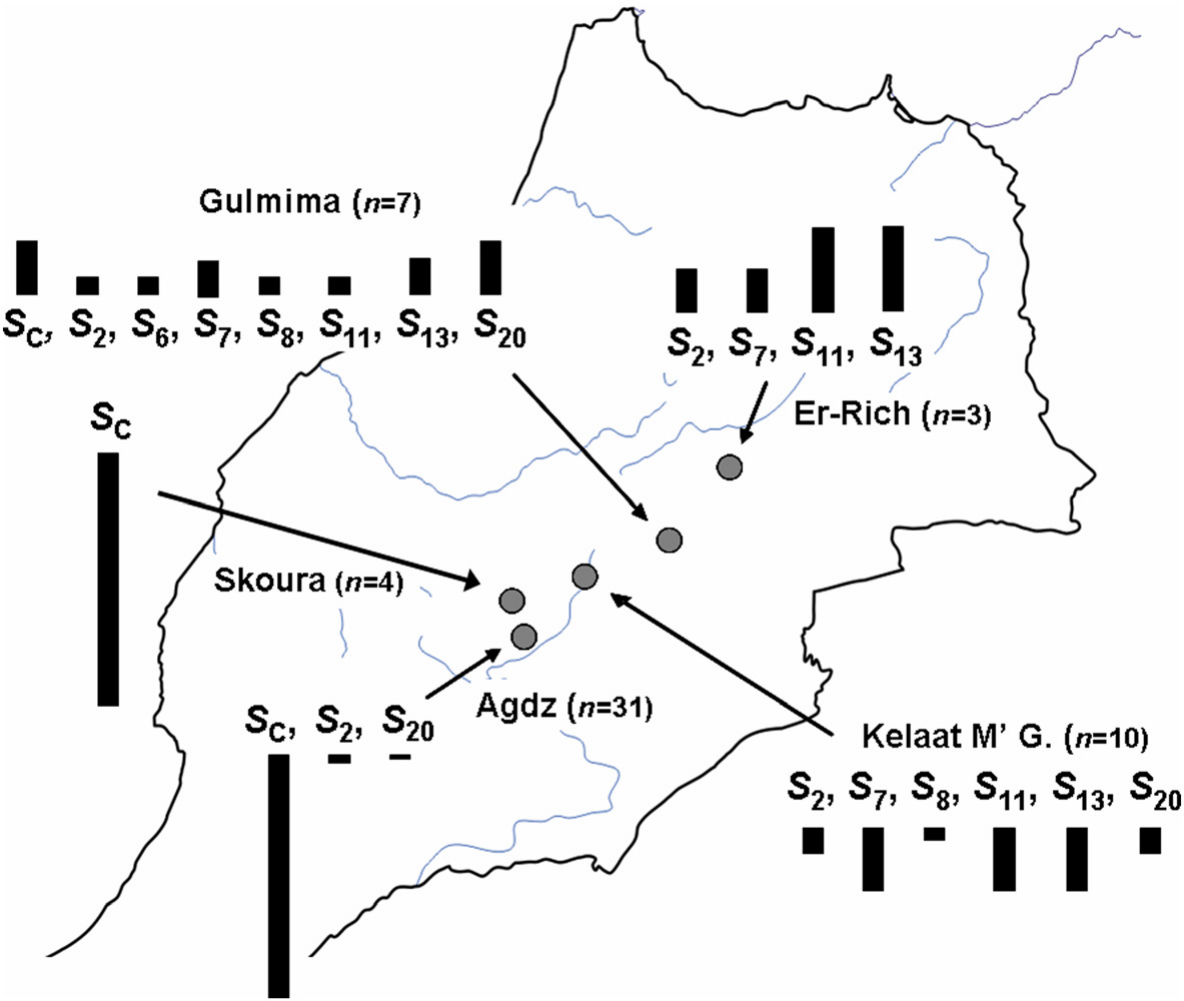
**The distribution and frequency of apricot *S*-alleles in Morocco.** The lengths of the black columns are proportional to the percentage frequency of specific *S*-alleles in a region (marked by grey spot), *n* is the number of sampled trees in each of the agro-ecosystems.

When completing the *S*-genotype of all the studied apricots (Table 1), seven previously described *S*-alleles were identified among the Moroccan genotypes. *S*_C_ was the most frequent *S*-allele in the tested Moroccan germplasm (occurred in 37 genotypes), followed by *S*_13_ (in nine), *S*_7_ and *S*_11_ (in eight), *S*_2_ and *S*_20_ (in six), *S*_8_ (in two) and *S*_6_ (in only one). The frequency and distribution of the *S*-alleles identified in the present study differed among the oases (Figure 4). In Tighnit and Armed oases (Agdz), almost all (94%) the 31 genotypes were homozygous for the *S*_C_-allele (Table 1). In these oases just three known *S*-alleles (*S*_2_, *S*_20_ and *S*_C_) were detected and allele frequency of *S*_C_ was 95% (Figure 4). In Skoura oasis, all genotypes were self-compatible and homozygous for the *S*_C_-allele. However, in Kelaat M’Gouna oasis six alleles (*S*_2_, *S*_7_, *S*_8_, *S*_11_, *S*_13_ and *S*_20_) were detected and all the genotypes were self-incompatible, carrying different combinations of the SI alleles with *S*_7_, *S*_11_ and *S*_13_ being the most frequent alleles (Table 1), each of them had an allelic frequency of 25% (Figure 4). In Gulmima oasis, eight known alleles (*S*_2_, *S*_6_, *S*_7_, *S*_8_, *S*_11_, *S*_13_, *S*_20_ and *S*_C_) were detected including the *S*_C_-allele, and *S*_20_ and *S*_C_ were the most frequent (21%) alleles (Figure 4). In Gulmima, only three genotypes were self-compatible, all of them heterozygous for the *S*_C_-allele. In Rich location (a mountain ecosystem), four alleles (*S*_2_, *S*_7_, *S*_11_ and *S*_13_) were found in only three self-incompatible genotypes.

Apricot trees are grown in oasis agro-ecosystems to provide shade under palm trees and the fruit is mainly used as livestock feed [31]. In these systems, apricot was propagated by seeds generally obtained from genotypes characterized by high productivity. Since self-compatibility generally leads to more reliable fruit set, the local population in Tighnit and Armed (Agdz) as well as Skoura oases (Figure 4) mostly propagated seeds from self-compatible and supposedly self-pollinated trees (based on the high allelic frequency of *S*_C_), thus leading to the loss of genetic diversity in the *S*-locus in those oases and the accumulation of the *S*_C_-allele. The apricot trees in these oases are generally less vigorous and younger than those from other locations, indicating that propagation was more active in the oases of southern Morocco than those in the north, probably due to more favourable climatic conditions. In Rich, Gulmima and Kelaat M’Gouna (Figure 4), the climatic conditions are generally unfavourable for fruit setting because of spring frost damage during the flowering period, contrary to Tighnit, Armed and Skoura oases with mild winters. Thus, apricots were not intensively propagated in Rich, Gulmima and Kelaat M’Gouna and hence the selection force for reliably setting (self-compatible) genotypes has been weaker over time. This might be an explanation for the higher number of *S*-alleles in these regions as compared with other oases. Bourguiba et al. [5], using SSR molecular markers, found that the genetic diversity of apricot in the Draa valley, a geographical region including Tighnit and Armed oases, was lower than in the Moulouya valley (situated further north). The presence of self-compatibility and human selection for higher yielding genotypes has resulted in the predominance of self-compatible genotypes in the Draa valley, southern Morocco, thus confirming the loss of genetic diversity.

### Crop evolutionary history

The Moroccan apricot genotypes belong to the Mediterranean group, considered to have originated from the Irano-Caucasian gene pool [4], being classified into a separate group (Mediterranean) by Hagen et al. [32]. The evolutionary and propagation studies in the Mediterranean region suggested that apricot was diffused through two main routes. Apricot was firstly dispersed through the northern shore of the Mediterranean Sea and a second dissemination route probably went through North Africa due to the Arabs [33-35]. This hypothesis has been recently confirmed by Bourguiba et al. [36] using SSR molecular markers to evaluate the genetic diversity of apricot in the Mediterranean area. The determination of the *S*-alleles in local Moroccan apricots has also allowed elucidating and confirming some aspects of crop evolution and propagation history of apricot in the Mediterranean Basin. Halász et al. [17], based on historical records and common alleles found in two germplasm, concluded that the Turkish germplasm strongly contributed to the formation and the genetic diversity of the Hungarian apricots. Additionally, the *S*_C_-allele might have been evolved somewhere south-east of Central Turkey [37], being later disseminated from there to the Mediterranean Basin and later to America, because in China and Central Asia, the centres of origin of apricot, all cultivars and genotypes are self-incompatible [15,16].

The presence of the *S*_C_-allele in the Moroccan apricots raises the question if this allele was introduced from Spain or by the Arabs through North Africa. The *S*_C_-allele was identified in the Spanish cultivar ‘Canino’ (*S*_2_*S*_C_) [12] that had been introduced in Morocco during the last century and propagated mainly by grafting, but also by seed in some regions. Bourguiba et al. [38] found evidence for gene flow between introduced cultivars and the local North African gene pool. As a consequence, the *S*_C_-allele might have transmitted from this cultivar to the local apricot population. However, the *S*_2_-allele in ‘Canino’ is also a mutated allele that confers self-compatibility, and hence it should have been inherited with the same frequency as *S*_C_. Since apricot in the Moroccan oases was selected based on its productivity, both alleles faced with the same selection pressure and therefore their frequencies should be similar in those regions. However, the *S*_2_-allele occurred in only six of the 55 tested accessions whereas the *S*_C_ was found in 37 of the 55 accessions and 33 were homozygous for the *S*_C_-allele (which means a double frequency of this allele). The discrepancy between the 70 occurrences of the *S*_C_-allele compared with only 6 occurrences of the *S*_2_ in Morocco indicates that *S*_2_ may not code for self-compatibility. Indeed, Gulmim-4 (*S*_2_*S*_7_) was confirmed to be self-incompatible based on fruit set ratio and pollen tube growth analysis after self-pollination [30]. All these facts seem to rule out the possibility that the *S*_C_-allele was introduced in Morocco through the Spanish cultivar ‘Canino’.

Furthermore, Halász [27] reported that the presence and frequency of *S*-alleles differ from China to Western Europe with some alleles exclusively occurring in specific geographic areas. For example, alleles *S*_10_-*S*_14_ were only detected in apricots from Armenia [14], while alleles *S*_8_, *S*_9_, *S*_19_ and *S*_20_ were detected in Hungarian apricots [24,27] but only in cultivars of putative Turkish origin [17]. The *S*_C_-haplotype originated from the *S*_8_-haplotype with a mutation in the pollen part leading to a non-functional *SFB*. The *S*_8_-allele has been detected in Central Europe and Turkey but not in the western European cultivars [17]. Since both the *S*_8_- and *S*_C_-alleles were detected in the Moroccan apricots, our results support their introduction to Morocco from the Irano-Caucasian group through North Africa and not from Spain. Halász et al. [17] also reported that the *S*_11_- and *S*_13_-alleles were detected in the southern part of Turkey, a region close to the ancient Phoenicia. In addition, these alleles were not detected in western and southern Europe but they were among the most frequent alleles in Moroccan apricots. The rare *S*_20_-allele was only identified in one Hungarian cultivar and in two Turkish landrace cultivars, but in our work this allele occurred in six local Moroccan genotypes. In addition, *S*_C_, *S*_8_, *S*_11_ and *S*_13_ were also detected in local Tunisian cultivars [39]. All these surprising results gave strong support to the Irano-Caucasian origin of the apricots from the oasis agro-ecosystems [4] and their introduction through North Africa [33,34].

**Figure 5:**
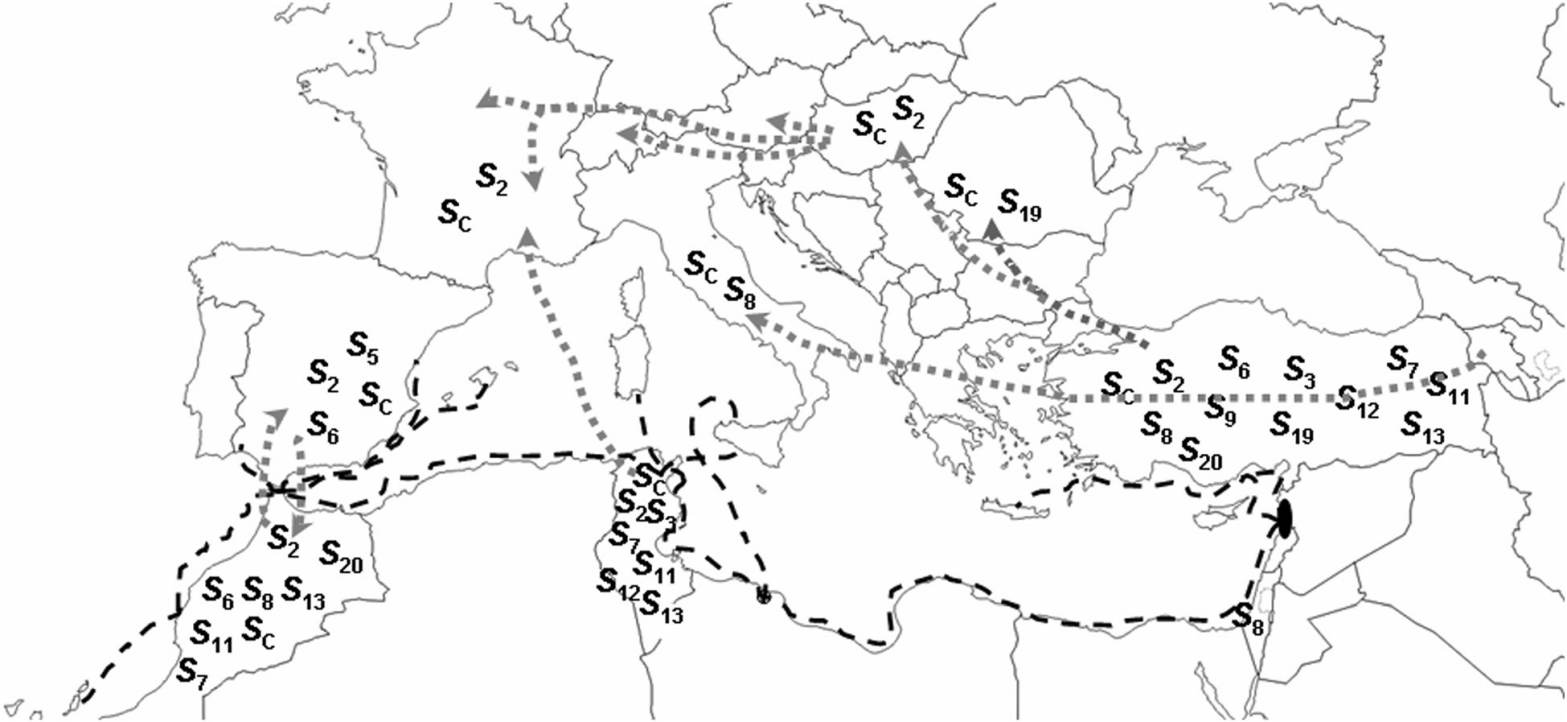
**Spread of the *S*-*RNase *alleles throughout the Mediterranean Basin.** Broken black lines indicate Phoenician shipping routs and putative primary dissemination of apricot in the Mediterranean Basin. Spotted grey arrows show some more recent documented movements among apricot germplasm based on Faust et al. [34]. *S*-alleles are shown based on published data by Alburquerque et al. [12]; Halász et al. [14,17,24]; Lachkar et al. [39]; Vilanova et al. [13] and the present study.

The spread of the identified *S*-alleles supports that the original introduction of apricot to the Mediterranean Basin was by Phoenician merchants, primarily to North Africa where this plant could be easily adapted (Figure 5). It is confirmed by the fact that from the 12?*S*-alleles identified in Turkey, seven were detected in Tunisia [39] and eight in Morocco (this study). The allelic richness might be even higher considering that only a limited number of accessions were used for the analysis (ranging from 12 to 55 accessions). A similar detailed study is not available for French and Italian apricot *S*-genotypes, but most of them are self-compatible [40]. Hence, a high frequency of SI alleles is not expected.

Following the Phoenician trade routs, apricots might have been introduced to Southern Europe. Apricot probably arrived to Italy directly from Armenia during the first century BC [34]. Then apricot spread to all the Roman empire, including Pannonia (Hungary). However, the most considerable upturn was a secondary introduction from Turkey to Hungary during the 16^th^ century [34]. The best Hungarian cultivars have Turkish origin, as confirmed by shared *S*-alleles [17] and linguistic evidence [34]. These cultivars were widely disseminated throughout northern Europe providing putative offspring such as ‘Luizet’ and ‘Nancy’. These cultivars were grown in northern France whereas those introduced by the Arabs were grown in the southern regions. All cultivars spread in northern regions are self-compatible [24], which must have been an important trait in environments with cold and wet springs. The inactivity of pollinating insects in such regions also imposed a selection pressure with similar consequences than for peach evolution [41].

North Africa seems to preserve much higher variability of apricot than Europe. In the present study, we have detected eight *S*-alleles already described, a low number as compared to those reported in Chinese genotypes (at least 52?*S*-alleles) (reviewed in Halász et al. [42] or Turkish germplasm (12 known *S*-alleles and four unknown alleles) [17]. However, most European cultivars are self-compatible [24] and only *S*_C_ and *S*_2_-alleles were detected in many cultivars, since *S*_8_, *S*_9_, *S*_19_ and *S*_20_ were detected only in the Hungarian cultivars of Turkish origin and their presence must be due to a secondary and more recent introduction from Turkey. This indicates that human selection might have had an immense contribution to the evolution and dissemination of the self-compatible phenotypes in countries north of the Mediterranean sea [43].

A very similar phenomenon may have taken place in some Moroccan oases, where a severely restricted number of *S*-alleles is found as compared to the whole allele pool in the country. This decrease in the number of *S*-alleles could be explained by the seed propagation of the self-compatible genotypes to increase reliable fruit set and productivity in oasis agro-ecosystems, giving rise to homozygous self-compatible genotypes (Table 1). This might have resulted in the reduced genetic diversity of apricot in Morocco indicated by Bourguiba et al. [5] based on SSR markers. The same fact was observed in Central Europe, where only a limited number of *S*-alleles was detected in landrace cultivars, most of them self-compatible [24] and showing a marked decrease in genetic diversity [44]. In consequence, the loss of genetic diversity might be explained by the occurrence of self-compatibility in apricot and the length of time that apricot has spent with the inbreeding ability in an environment without its wild relatives such as the Moroccan oases or Central Europe.

## Conclusions

The *S*-genotypes of 55 Moroccan apricot accessions were determined, detecting an unexpected great variability in the *S*-locus. In addition, many of these *S*-alleles were previously thought to be present only in the Irano-Caucasian region. The presence of these alleles in North Africa indicates that this region received and preserved more variability from the Irano-Caucasian than from the European gene pool. However, the occurrence of self-compatibility in isolated environments such as several oases and the great selection pressure for high yielding genotypes resulted in the accumulation of the *S*_C_-allele, self-compatibility becoming predominant in such locations. This evolutionary trend is very similar to that faced by apricot in northern Europe. The European and Moroccan germplasm might have originated from different genetic bases, but reached a similar stage over time: the continuous pressure for self-compatibility by growers resulted in a serious loss of genetic diversity. Our results deepen the knowledge on apricot crop history and highlight the genome shaping force of the sexual reproduction strategy in *Prunus*.

## Methods

### Plant material

This study was carried out in four different locations of southern Morocco with wealthy apricot genetic resources: Agdz oasis in Draa valley, Skoura oasis in Dades valley, Goulmima oasis in Ziz valley, and Er-Rich valley in the Ziz region of the high Atlas mountains. A total of 55 local genotypes from different zones of each region were included in the present study (Table 1). Trees in agro-ecosystems were sampled according to the results of a preliminary screening for valuable traits in Moroccan apricots. In all regions, apricots were collected from different parts of the specific agro-ecosystems. Some previously *S*-genotyped cultivars (‘Ceglédi Piroska’ , ‘Korai zamatos’) were used as control [14,24].

### DNA extraction

Genomic DNA was extracted from fully expanded young leaves using a DNeasy Plant Mini Kit (Qiagen, Hilden, The quantity and quality of DNA were analyzed by NanoDrop™ ND-1000 spectrophotometer (Bio-Science, Budapest, Hungary).

### Genomic PCR with *S-RNase* and *SFB*-specific primers

PCR was conducted according to Sutherland et al. [28] using the degenerate primers EM-PC2consFD and EM-PC3consRD for the amplification of the second intron region of the *S-RNase* gene. To amplify the first intron, the fluorescently labeled (_JOE_) forward primer SRc-F [10] was used in combination with the reverse primer SRc-R [13].

For the identification of the *S*_C_-haplotype, a two-step approach was used. An allele-specific reverse primer, AprSC8R was designed by Halász et al. [17] to amplify the *S*_C_/*S*_8_-*RNase* allele and used in combination with PaConsI F [45]. The amplification was carried out using a temperature profile with an initial denaturing of 94°C for 2 min, 35 cycles of 94°C for 30 s, 55°C for 1.5 min and 72°C for 2 min, and a final extension of 72°C for 5 min. *SFB*_C_/*SFB*_8_-specific primers, AprFBC8-F and AprFBC8-R were designed based on the V2 and HVb variable region of the *SFB*_C/8_ allele [17]. The amplification was carried out as described for the *S*_C_/*S*_8_-*RNase* specific primers.

PCR was carried out in a PTC 200 thermocycler (MJ Research, Budapest, Hungary). For amplification of the *S*-*RNase* first and second introns, we used the programs originally described for the primers [13,28]. Approximately 20–80 ng of genomic DNA were used for PCR amplification in a 25-μL reaction volume, containing 1?×?PCR buffer (Sigma, Budapest, Hungary) with final concentrations of 10 mm Tris-HCl (pH 8.3), 50 mm KCl, 1.5 mm MgCl_2_, 0.2 mm of dNTPs, 0.4 μm of each primer and 0.625 U of *Taq* DNA polymerase (Sigma, Budapest, Hungary). Using the second intron sequence information, an allele-specific forward primer, PaS20-F (5’ CCTTTGGGTATGCTAGATGAAA 3’) was designed to selectively detect the *S*_20_-allele and it was used in combination with the reverse EM-PC5consRD primer [28]. An allele-specific restriction enzyme for *S*_13_ was chosen using the TACG program at Biology WorkBench (http://seqtool.sdsc.edu). The *PstI* enzyme was used after amplification for the second intron region of allele *S*_13_.

### Data evaluation

The PCR products were separated on 2% TAE agarose gels at 100 V for 2 h and DNA bands were stained with ethidium bromide. Fragment sizes were estimated by comparison with the 1 kb?+?DNA ladder (Promega, Madison, Wis.). For exact size determination of *S-RNase* first intron region fragments smaller than 500 bp, the fluorescently labeled products were run in an automated sequencer ABI PRISM 3100 Genetic Analyzer (Applied Biosystems, Budapest, Hungary) using the GENOTYPER 3.7 software and GS500 LIZ size standard (Applied Biosystems, Budapest, Hungary). The frequency of *S*-alleles in specific agro-ecosystems was calculated by dividing the number of copies of a particular allele by the number of copies of all alleles, and was expressed in percentage.

## Competing interests

The authors declare that they do not have competing interests.

## Authors’ contributions

OK designed the experiments, carried out part of the molecular genetic studies, and drafted the manuscript. AH participated in data interpretation and critically revised the manuscript. RSC participated in the design of the study and critically revised the manuscript. JH conceived of the study, participated in molecular genetic analysis, prepared the illustrations and helped to draft the manuscript. All authors read and approved the final manuscript.
